# Bacteriophage Treatment before Chemical Disinfection Can Enhance Removal of Plastic-Surface-Associated Pseudomonas aeruginosa

**DOI:** 10.1128/AEM.00980-21

**Published:** 2021-09-28

**Authors:** Elyse Stachler, Anina Kull, Timothy R. Julian

**Affiliations:** a Eawag, Swiss Federal Institute of Aquatic Science and Technology, Dübendorf, Switzerland; b Swiss Tropical and Public Health Institute, Basel, Switzerland; c University of Basel, Basel, Switzerland; University of Manchester

**Keywords:** biofilm, phage therapy, opportunistic pathogens

## Abstract

Opportunistic pathogens can linger on surfaces in hospital and building plumbing environments, leading to infections in at-risk populations. Furthermore, biofilm-associated bacteria are protected from removal and inactivation protocols such as disinfection. Bacteriophages show promise as tools to treat antibiotic-resistant infections. As such, phages may also be useful in environmental applications to prevent newly acquired infections. In the current study, the potential of synergies between bacteriophage and chemical disinfection against the opportunistic pathogen Pseudomonas aeruginosa was assessed under various conditions. Specifically, surface-associated P. aeruginosa was treated with various concentrations of phages (P1 or JG004), chemical disinfectants (sodium hypochlorite or benzalkonium chloride), or combined sequential treatments under three distinct attachment models (spot inoculations, dry biofilms, and wet biofilms). Phages were very effective at removing bacteria in spot inoculations (>3.2 log_10_ removal) and wet biofilms (up to 2.6 log_10_ removal), while phages prevented the regrowth of dry biofilms in the application time. In addition, phage treatment followed by chemical disinfection inactivated P. aeruginosa cells under wet biofilm conditions better than either treatment alone. This effect was hindered when chemical disinfection was applied first, followed by phage treatment, suggesting that the additive benefits of combination treatments are lost when phage is applied last. Furthermore, we confirm previous evidence of greater phage tolerance to benzalkonium chloride than to sodium hypochlorite, informing choices for combination phage-disinfectant approaches. Overall, this paper further supports the potential of using combination phage and chemical disinfectant treatments to improve the inactivation of surface-associated P. aeruginosa.

**IMPORTANCE** Phages are already utilized in the health care industry to treat antibiotic-resistant infections, such as those on implant-associated biofilms and in compassionate-care cases. Phage treatment could also be a promising new tool to control pathogens in the built environment, preventing infections from occurring. This study shows that phages can be combined effectively with chemical disinfectants to improve the removal of wet biofilms and bacteria spotted onto surfaces while preventing regrowth in dry biofilms. This has the potential to improve pathogen containment within the built environment and drinking water infrastructure to prevent infections by opportunistic pathogens.

## INTRODUCTION

Infections due to opportunistic pathogens (OPs) (such as Pseudomonas aeruginosa, Legionella pneumophila, and nontuberculous mycobacteria) are on the rise and result in up to $45 billion in direct health care costs annually in the United States (1). OPs, often members of the natural environment and/or human flora, typically do not cause disease in healthy individuals but can be problematic for at-risk populations, such as those in nursing homes and hospitals. Hospital-acquired infections (HAIs) from OPs represent a growing risk to patients and can negatively affect patient outcomes (2, 3). Additionally, these infections are increasingly resistant to antibiotics (2, 3). In 2017, P. aeruginosa alone was responsible for 32,600 multidrug-resistant HAIs in the United States, resulting in 2,700 deaths (4).

OPs are readily transmitted via the built environment. In hospitals, approximately 7.6% of patients in high-income countries and 15.5% in low-income countries will acquire a secondary bacterial infection (5, 6). Patients are particularly prone to acquiring a secondary infection in the hospital when they are placed in a room where the previous patient had the infection (7). Environmental cleaning and disinfection are important parts of an infection control strategy; however, pathogens can linger on surfaces in both wet and dry biofilms and can rapidly recolonize disinfected surfaces due to personnel interactions (8–12). Surfaces are now recognized as an important source of infection (12), with one study finding dry biofilms contaminating all studied surfaces in a hospital environment (9). Dry biofilms can regrow when coming into contact with nutrients and moisture, the source of which can be the cleaning products themselves (13–15). In addition, bacteria can acquire resistance to disinfectants similarly to the acquisition of resistance to antibiotics, through repeated exposure to subinhibitory concentrations (16, 17). Importantly, resistance to disinfectants can be correlated with antibiotic resistance and vice versa (18–21).

OPs can also be a threat through the building plumbing environment (22, 23), where they can live in biofilms and slough off where individuals can be exposed through the aerosolization of the OPs through activities such as showering, handwashing, and toilet flushing. Currently, best practices for eliminating the growth of these pathogens require on-site treatment such as additional disinfection, heat shock treatment, or an increased boiler temperature (24). However, these practices can be costly, require additional maintenance, and can select for different pathogens or allow more resistant subpopulations of pathogens to later populate the system (24). For both water and surface disinfection, there is a clear need for novel methods for controlling pathogens in the built environment.

Bacteriophages have received increased interest in recent years as alternative treatment strategies to combat the antibiotic resistance crisis. Most research into phages as antibacterial agents has focused on applications such as using phages to treat bacterial infections in patients due to antibiotic-resistant bacteria (25, 26). Less attention has been paid to the opportunity to use phages as biocontrol agents in the environment (27). Yet, increasingly, phages are being used in other industries such as for medical devices (28), agriculture (29), aquaculture (29–31), and the food industry (29, 32) as prophylactic treatments to prevent disease. However, challenges remain in the widespread implementation of phage treatments, including the development of phage resistance, regulation of phage-based products, and unknown environmental consequences of phage treatment (33). While there are many challenges, there are also numerous benefits of phage treatment, including better control and penetration of biofilms (34), resensitization of antibiotic-resistant bacteria (35), and the ability to evolve to evade bacterial defenses (36).

These benefits make phages promising agents for pathogen control in the built environment, such as disinfection agents against OPs. However, it is unclear how phages may interact with chemical disinfectants that are also used in these environments, either for use in beneficial combinations to improve inactivation or in the presence of residual levels of chemicals that may interfere with phage effectiveness. In contrast, there has been substantial interest in investigating the combination of phage and antibiotic treatments (37–44). Phage and antibiotic combination treatments enhance biofilm removal relative to either treatment alone (38, 41, 44), prevent resistance development (39, 41), and allow antibiotics to be effective at lower concentrations (38–40, 42). It has also been shown that the treatment order can affect the outcome, with phage pretreatment before antibiotic treatment enhancing removal more than antibiotic pretreatment or even simultaneous treatment (41, 42, 44). In contrast, less attention has been given to the interplay of phages and chemical disinfectants for use in combination treatments. Previous research has looked at simultaneous treatment with phage and disinfectants, with varying success (45–48), likely due to the confounding of the two treatments by the inactivation of the phages by the disinfectants. Also, most studies have shortcomings in the thoroughness of testing of a range of concentrations and a small dynamic quantification range since they determine removal by crystal violet staining or optical density (OD) measurement (46–49), which limits the amount of inactivation that can be seen.

The current study looks at the interplay between phage treatment and chemical disinfection to understand how these treatments could be used in sequential treatments to improve pathogen removal associated with surfaces. Models for wet biofilms, dry biofilms, and spot inoculations were investigated. Tests were conducted to evaluate each treatment type individually as well as after a combination of sequential treatments. Additionally, the effect of the order of the treatments was investigated, as was the effect of the chemical disinfectants on the phages themselves.

## RESULTS

### Spot inoculations.

**FIG 1:**
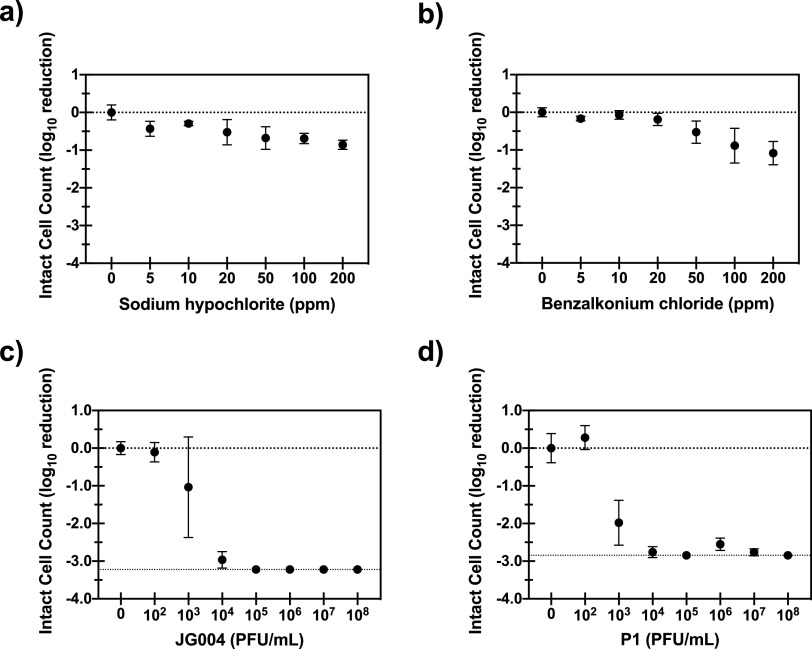
Log reduction of P. aeruginosa in spot tests. The data represent the averages and standard deviations from four replicates of the log_10_ reduction of the intact-cell counts of P. aeruginosa treated with sodium hypochlorite (a), benzalkonium chloride (b), phage JG004 (c), or phage P1 (d). The dotted lines at the bottom of panels c and d represent the lower limit of quantification (LLOQ) of the FCM measurements. The applied phage concentrations correspond to multiplicities of infection (MOIs) of approximately 2 × 10^−5^ to 20.

Phages were effective at inactivating bacteria spotted onto plastic surfaces and much more effective than chemical disinfectants for the tested parameters. Sodium hypochlorite achieved a 0.29 to 0.86 log_10_ removal value (LRV) for concentrations (initial intact-cell count [ICC] of approximately 10^5^ bacteria per slide) ranging from 5 to 200 ppm (Fig. 1a), while benzalkonium chloride achieved a 0.07 to 1.1 LRV (Fig. 1b). Phage treatment resulted in a 1.0 to >3.2 LRV for phage JG004 (Fig. 1c) and a 2.0 to >2.9 LRV for phage P1 (Fig. 1d), representing multiplicities of infection (MOIs) from 2 × 10^−5^ to 20. Combination experiments were not able to be conducted with the spot inoculation setup since the lower limit of quantification (LLOQ) of the flow cytometer (FCM) was reached even with a very low phage MOI (2 × 10^−3^). This is partially due to the lower concentration inoculated onto each slide for the spot tests as well as the greater effect of phages on the more readily available spotted bacteria than on bacteria living in biofilms.

### Dry biofilms.

**FIG 2:**
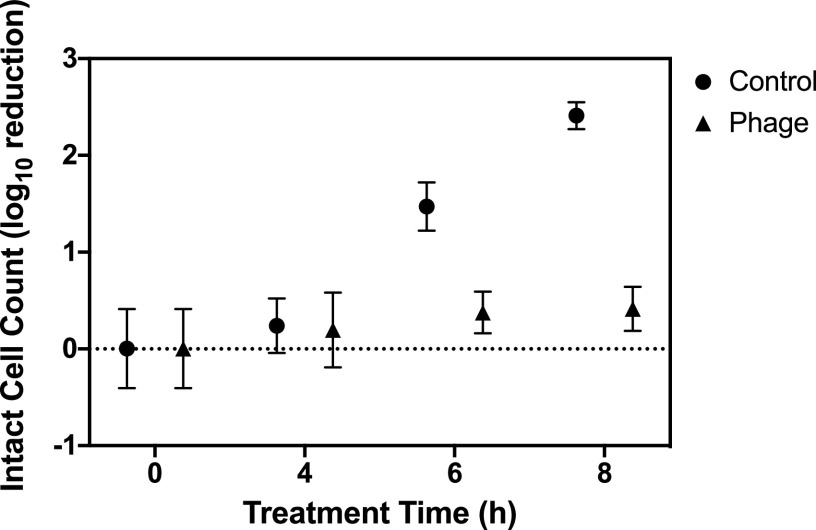
Log reduction of P. aeruginosa in dry biofilms after rehydration. The data represent the averages and standard deviations from four replicates of the log_10_ reduction of the intact-cell counts of P. aeruginosa treated with 10^6^ PFU/ml of phage P1 for various treatment times compared with no-treatment control biofilms.

In the dry biofilm setup, 24-h biofilms were dried in a biohood for 2 h before treatment with either phage or sodium hypochlorite. Phage treatment mixtures and controls were seeded in nutrient-rich broth to simulate a product that would include nutrients to allow efficient phage infection. Sodium hypochlorite inactivated bacteria in a range similar to that with the spot inoculations, exhibiting a 0.16 to 0.58 LRV with concentrations ranging from 5 to 200 ppm (initial ICC of approximately 10^5^ bacteria per biofilm). In contrast to the spot inoculation, however, initial tests of phage treatment of dry biofilms (incubation time of 4 h) resulted in essentially no removal of the biofilm, regardless of the phage concentration. To explore this phenomenon further, growth curves of cells recovered from biofilms before and after drying for 2 h were established to explore the growth properties of the two applied conditions (see Fig. S1 in the supplemental material). No significant difference was found for the carrying capacity, initial population size, or growth rate of the two populations. However, there was a statistically significant (*P* < 0.01) difference in the lag times between the two populations (3.06 ± 0.17 h for undried biofilms compared to 6.97 ± 0.13 h for dried biofilms). Bacteriophages need actively growing hosts for efficient infection (50); therefore, it is plausible that the effectiveness of phage treatment is reduced on dry biofilms because P. aeruginosa cells have entered a dormant growth phase, which cannot support efficient phage infection and replication, particularly if the treatment duration (here, 4 h) is shorter than the lag phase (estimated here at ∼7 h). To test this hypothesis, additional dry biofilm experiments were conducted with phage P1 for longer application times (up to 8 h). Figure 2 shows that even though the positive controls grew without phage challenge, the presence of phages prevented the regrowth of the dry biofilms, although they did not work to remove the already existing dry biofilm. An additional experiment investigating the effect of the phage concentration on the extended treatment (Fig. S2) showed that phage could potentially result in some removal of dry biofilms; however, the removal was independent of the phage concentration due to the length of the application (*P* > 0.59 for 10^1^ to 10^9^ PFU/ml).

### Wet biofilms.

**FIG 3:**
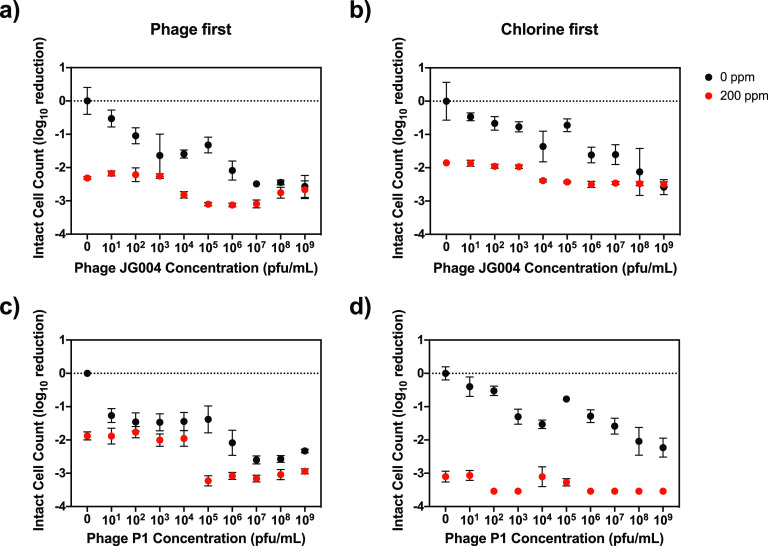
Log reduction of P. aeruginosa in wet biofilms by phage and chlorine. The data represent the averages and standard deviations from five replicates of the log_10_ reduction of the intact-cell counts of P. aeruginosa in wet biofilms due to various sequential treatments with phages and sodium hypochlorite compared with phage-only and chemical-only treatment control biofilms: phage JG004 followed by chlorine (a), chlorine followed by phage JG004 (b), phage P1 followed by chlorine (c), and chlorine followed by phage P1 (d). Phages were used at concentrations of 10^1^ to 10^9^ PFU/ml (corresponding to multiplicities of infection of 10^−6^ to 10), while sodium hypochlorite was used at 200 ppm.

In the wet biofilm setup, both phage and chemical disinfection treatments were effective at inactivating the bacteria in the biofilms, with disinfection alone leading to a 2 LRV, while phage treatment alone reached a 2.6 LRV (initial ICC of approximately 10^6^ to 10^7^ bacteria per biofilm). However, a >3 LRV could be achieved only with combined treatment. In combination experiments, more bacteria were inactivated when phage pretreatment was followed by sodium hypochlorite (200 ppm) treatment than with either treatment alone (Fig. 3a and c). This effect is seen for phage concentration levels at or above approximately 10^5^ PFU/ml, equivalent to an MOI of approximately 0.01. With both phages, the LRV of the highest applied phage-only treatment (MOI = 10) is achieved with a much lower MOI of 0.01 plus chlorine. In addition, this removal is significantly greater than that with chlorine alone. At chlorine concentrations lower than this (5 and 20 ppm), phage treatment dominates (Fig. S1).

The order of the applied treatments was also reversed, with sodium hypochlorite application being followed by phage treatment. With phage JG004, an increase in removal is also seen with phage concentrations of over 10^5^ PFU/ml; however, this benefit is less pronounced than when phage treatment is applied first. For phage P1, the chlorine treatment dominates over the entire concentration range of phages applied. To investigate this further, this application order was also tested with lower chlorine concentrations of 5 and 20 ppm (Fig. S3). For 20 ppm, chlorine also dominates over the entire range of phage concentrations, with phage treatment offering no additional benefit. For 5 ppm, a small added benefit is seen for phage concentrations of over 10^6^ PFU/ml; however, this results in lower LRVs than with phage treatment on its own.

### Inactivation of phages by disinfectants.

To understand how phages may interact with chemical disinfectants in the environment, phage inactivation by the disinfectants used in this study was evaluated (Fig. S4). Both phage JG004 and phage P1 are readily inactivated by sodium hypochlorite, reaching the limit of detection (>7 LRV) with CT (concentration × time) values of 15 and 50, respectively. In contrast, the phages were more resistant to inactivation by benzalkonium chloride (BAC). Both phages were stable up to a CT value of 20, where phage JG004 reached a 1.6 LRV that did not increase further up to a CT value of 200, while phage P1 reached a 0.23 LRV at a CT value of 20, which linearly increased to a 4.7 LRV at a CT value of 200.

## DISCUSSION

### Phages are effective at removing surface-associated bacteria.

While this paper sought to uncover the potential benefits of combined treatment with phages and disinfectants, a clear result from this paper is the effectiveness of phage treatment on its own for certain attachment models. Specifically, combination treatment of spotted bacteria was not possible due to the effectiveness of phage treatment at MOIs as low as 10^−3^ combined with the limit of quantification of the methods used. Many other studies have looked at phage treatment of spotted bacteria and wet biofilms, with similar results (49, 51–53). A Salmonella phage mixture at an MOI of 10 effectively reduced Salmonella dried on glass and steel surfaces by 2.1 to 4.3 log_10_ units, which corresponded to a >99% removal rate (51). Similarly, Escherichia coli cells dried on stainless steel, ceramic, and polyethylene were successfully treated with bacteriophage mixture BEC8. With testing at room temperature at an MOI of 100, E. coli cells were reduced by 5 log_10_ units within 2 to 4 h (52). D’Accolti et al. reported the efficient removal of wild-type drug-resistant Staphylococcus aureus, E. coli, and P. aeruginosa isolates collected from hospital surfaces on ceramic, plastic, and glass test surfaces. Relatively high MOIs of 10^3^ were applied and reduced the spread of 100 CFU on the surfaces by over 90% in 1 h (53). In comparison, our study found that the phage managed the removal of approximately 3 log_10_ units at a low MOI of 0.1 within 4 h, further demonstrating the utility of phage as a treatment for bacteria on surfaces. In wet biofilms, Alves et al. found a >95% reduction in P. aeruginosa static biofilms treated with a cocktail of six phages at an MOI of 10 for 4 h (54), while Zhang and Hu found ∼75% biofilm removal for an MOI of 1 after 72 h (49). Comparatively, the current study found >99% biofilm removal at a similar MOI for monophage treatments lasting 4 h. However, it has also been demonstrated that phage can have the opposite effect and actually promote the growth of biofilms at low concentrations (55). While the phages used in this study were effective at inactivating biofilms at very low MOIs, this emphasizes the importance of phage selection and characterization for future product formulations.

### In the current study, phage and chemical disinfectants can be used together to increase removal.

This study found that the use of sequential treatment with phages and chemical disinfectants could result in greater removal of biofilms than either treatment alone. Importantly, combining lower concentrations of phages with chemical disinfectants could reach the same level of inactivation as that of the highest tested phage concentrations alone. This could ease the burden of scaling up phage production for further products built on this principle. In addition, previous research has shown that the use of lower concentrations of phage and antibiotics in combined treatments reduces the development of resistance to either treatment (39, 56). This phenomenon may also be applicable to sequential disinfectant and phage approaches, although this still needs to be validated. Previous research has mostly focused on comparing phage treatment alone to chemical disinfection alone. Fewer studies have looked at the combinatory effect. The simultaneous treatment of Listeria monocytogenes dried on surfaces with a quaternary ammonium compound (QAC) and *Listeria* phages resulted in the same removal efficiency as that with the QAC alone at higher concentrations (45). Agún et al. found that phage did not increase the removal of biofilms when added to chemical disinfectants, although that study also found some inactivating effect of the chemical disinfectant on the phages themselves (46). For combined treatment with sodium hypochlorite and phages, beneficial effects on wet biofilms were reported. Simultaneous treatment with a phage and sodium hypochlorite revealed additive effects as wet biofilms of Salmonella enterica serovar Typhimurium were completely removed, whereas the phage or chemical alone could not remove the biofilms completely (47). In contrast, the combined treatment of phage and benzalkonium chloride was no more effective in biofilm reduction than benzalkonium chloride alone (47). Zhang and Hu looked at the interactions of phage and sodium hypochlorite on the formation and removal of P. aeruginosa wet biofilms and reported synergistic effects in biofilm lysis (49). Another study found that adding Triton X-100 (a nonionic detergent) could enhance biofilm removal by an S. aureus phage, although it reached the same level of removal as that of phage alone after 48 h, as determined by OD measurements (48). However, the current study reports this interaction on wet biofilms thoroughly over a range of concentrations (difference of 9 orders of magnitude in MOIs), showing that significantly lower phage concentrations can be used in combination with a chemical disinfectant to achieve the same inactivation as that with higher phage concentrations alone.

In contrast, treatment of wet biofilms with sodium hypochlorite followed by phage reduced the impact of the phage treatment in the current study. Since the chemical disinfectant was physically removed and quenched before the application of phages, it is unlikely that this is due to the inactivation of the phages by the disinfectant. Applying chlorine first may make the cells enter a more protective, inactive state that inhibits efficient phage attachment and infection, reducing the effect of phage treatment. Alternatively, chlorine may damage the bacteria in the outer layer of the biofilm, preventing phage infection that can subsequently self-propagate deeper into the biofilm (57–59). This has important implications for how combined treatments may be applied as well as warning against potential interactions with disinfectant residuals that may be present in environments where phages are applied. Sukumaran et al. tested chemical disinfection sequentially followed by phage treatment on chicken filets and found that phage application resulted in an additional log unit of removal (60). However, Sukumaran et al. did not compare this treatment to phage treatment followed by disinfection.

### Environmental conditions greatly affect phage treatment.

In the present study, phages were incubated at the optimal temperature for bacterial growth in the host. This may lead to an overestimation of the effect of phage treatment on environmental systems, where the environmental conditions (such as temperature or humidity) may not be as ideal for phage infection. It was found that phage prevented the regrowth of bacteria in dry biofilms but could not necessarily remove the biofilms, which may be due to insufficient microbial activity of Pseudomonas aeruginosa. Phage infection efficiency depends on the growth rate of the host bacteria (50). This may help explain why no known studies have investigated phage treatment of dry biofilms, whereas there is extensive research on chemical treatment of dry biofilms (61–65). In environmental systems, many stressors are present and could potentially reduce phage infectivity. To overcome this, phages may need to be applied for longer times or may need to be applied in combination with nutrients to allow efficient infection.

### Phage treatment will be impacted by residual disinfectants.

In order for phage treatment to be a feasible option in environments where disinfectants are used, the order of treatment will impact effectiveness. If the phages and disinfectants are to be combined either simultaneously or sequentially, the effect of disinfectants on the phages themselves must be determined for three reasons. First, simultaneous application will be effective only if the phage is resistant to the disinfectant. Second, residual disinfectant concentrations lingering on surfaces or in the water environment may render phage treatment useless if the phage used is particularly sensitive to the disinfectant present. Finally, it may be beneficial to disinfectant systems after phage treatment to prevent large quantities of phage particles from being released into the environment unregulated, where it is uncertain how they may affect the natural microbiota. We found that even low sodium hypochlorite concentrations (5 and 20 ppm) would be high enough to inactivate the phage for the applied contact time (10 min). On the other hand, higher phage resistance to benzalkonium chloride (BAC) suggests that a combined BAC and phage formulation may be able to be applied simultaneously.

### Phage diversity is vast; it will be important to verify each phage individually for treatment use.

Studies on phage sensitivity to sodium hypochlorite demonstrate a wide range, with most phages exhibiting susceptibility (66–69) (as in the present study), while other phages are highly resistant to sodium hypochlorite, even with high concentrations and long exposures (800 ppm for 30 min) (66, 70). Similarly, results from other studies indicate no uniform effect of QACs on phages. Many studies demonstrate phages to be readily inactivated by QACs (46, 69), while others show phages to be resistant to all tested concentrations and exposure times, even at 10 times the recommended concentration (70, 71). Both phages in the current study are readily inactivated by sodium hypochlorite but are more resistant to inactivation by benzalkonium chloride. Furthermore, phage JG004 shows more resistance to BAC than phage P1. JG004 has been previously characterized and does not have an external envelope (72), which could explain this greater resistance, as it has been shown that enveloped viruses are more sensitive to QACs (73). This further illustrates the wide variation in phage susceptibility, and more research is needed in order to predict how phages may respond to chemical disinfection based on viral characteristics, especially because these products could be used in many environments with diverse chemical residuals. In addition, this emphasizes that individual phage formulations will need to be evaluated on a case-by-case basis for future product development.

### Limitations and future directions.

This study was conducted in a controlled laboratory setting to understand the interactions of phage and chemical treatments; thus, it can only limitedly inform how these treatments would behave in practical applications. More research will be necessary to translate this research for use in real-world settings, including looking at a range of environmental conditions, application times, sensitivities to residual chemicals, and nutrient formulations. However, this study begins to inform how to build combination experiments for practical testing, namely, by demonstrating how treatment order can influence applicability and that lower phage MOIs can be just as effective as higher MOIs when combined with chemical disinfection. In addition, this study evaluated single-species surface-associated communities. In real-world scenarios, many species of bacteria will be present, which will complicate treatment. Nonhost bacteria could impede the infection of target bacteria, while on the other hand, phages have been shown to “hitchhike” on carrier bacteria to more effectively reach their targets (74). Future research should investigate multispecies communities and how phage treatment is altered for target bacteria as well as how nontarget bacteria may be affected. This study demonstrated variability in phage performance and susceptibility to chemical disinfectants even when comparing two phages. Given the wide variety of phages active against P. aeruginosa, potential phage-based treatments should consider this breadth and investigate combining phage types for a more robust treatment regime. Finally, this study utilized flow cytometry to be able to quickly evaluate the inactivation of bacteria in samples, allowing high sample throughput. However, this led to a higher limit of quantification than could be achieved by more traditional culture-based methods. For applications that require showing higher log removal values, other methods of quantification may need to be considered.

Overall, the present study supports the potential use of phage-based treatment options as a strategy for the reduction or elimination of pathogenic bacteria in the built environment, including within biofilms, which are more resistant to traditional removal methods. In addition, combining chemical disinfection with phage treatment has the potential to result in greater removal of bacteria than with either treatment alone, although evidence suggests that the application order may be important to consider. Importantly, this study demonstrates that formulations with lower phage concentrations can reach the removal rates of higher phage concentrations when combined with chemical disinfection, a phenomenon that has been widely shown with antibiotics. This will make the scale-up of these formulations more feasible and potentially make it less likely that environmental bacteria will develop resistance to either treatment. Despite initial promising results, more research is warranted into the interaction of chemical disinfectants and phage-based treatments and their application in real-world settings.

## MATERIALS AND METHODS

### Bacterial and viral strains and growth conditions.

Pseudomonas aeruginosa strain PAO1 (DSM 19880, provided by Konstanze Schiessl and Martin Ackermann, Eawag) and bacteriophage JG004 (DSM 19871) were obtained from the Deutsche Sammlung von Mikroorganismen und Zellkulturen (DSMZ) (Braunschweig, Germany). Phage P1 was isolated from wastewater by ZnCl_2_ precipitation followed by a double-agar-layer assay with PAO1 as the host bacterium. PAO1 was grown in tryptic soy broth (TSB) (Sigma-Aldrich, St. Louis, MO, USA) and incubated overnight at 37°C for all experiments.

### Experimental setup.

To test the surface disinfection efficacy of integrated chlorine-based chemical disinfection and phage control, the opportunistic pathogen Pseudomonas aeruginosa was seeded onto surfaces under spot inoculation (representing surfaces or fomites), dry biofilm (representing communities on noncritical hospital surfaces) (8), and wet biofilm (representing building plumbing or medical devices) conditions. The spot inoculations and biofilms were then disinfected with phage treatment followed by chlorination, or chlorination followed by phage treatment, under various combinations of phage concentrations and chlorine concentrations. Intact-cell counts (ICCs) of P. aeruginosa were quantified using flow cytometry after treatment, and log_10_ removal values (LRVs) were calculated based on reductions relative to a no-treatment control.

To inoculate surfaces with biofilms, a culture of PAO1 grown overnight was diluted to an OD at 600 nm (OD_600_) of 0.05 (equivalent to approximately 10^7^ CFU/ml, confirmed by culturing) in LB medium (AppliChem, Darmstadt, Germany) and used for seeding in all experiments. For spot inoculation experiments, plastic coverslips were placed flat in the bottom of 12-well microtiter plates (Greiner Bio-One, Frickenhausen, Germany). Coverslips were prepared prior to experimentation by washing with 70% ethanol followed by irradiation with UV light for 20 min (Puritec HNS 30W G13 UV-C germicidal lamp; Osram, Munich, Germany). Next, 10 μl of the seed culture (equivalent to 10^5^ CFU) was spotted onto each test coverslip, while sterile LB medium was spotted for negative controls (*n*_spot_ = 2 per plate). The coverslips were dried in a biosafety cabinet for 30 min before treatment to ensure attachment to the surface.

For dry biofilms, plastic coverslips were prepared as described above for the spot inoculation experiments but inserted perpendicularly to the bottom of the well to be used as the surface for cell attachment and biofilm growth. Each well was seeded with 1 ml of the seed culture. Coverslips were trimmed prior to use to allow the lid of the microwell plate to close. For wet biofilms, 96-well microtiter plates (Nunc MicroWell microplates with a Nunclon Delta cell culture-treated surface; Thermo Scientific, Waltham, MA, USA) were seeded with 200 μl of the seed culture (equivalent to 2 × 10^6^ CFU) in each test well. The outermost wells on each plate were not used for experiments but were instead filled with nanopore water to eliminate evaporation effects on testing. Negative controls were included on each microplate by inoculating the wells with sterile LB medium instead of the seed culture (*n*_wet_ = 5 and *n*_dry_ = 2 per plate). Microplates were incubated at 37°C under static growth conditions for 24 h for biofilm growth. After 24 h, the unattached cells were removed from the wells, and biofilms were washed twice with phosphate-buffered saline (PBS). Wet biofilms proceeded directly to treatment, while dry biofilms were first placed under laminar flow conditions in a biohood to dry the biofilms for 2 h before proceeding to treatment.

### Treatment of surface-attached bacteria.

Biofilms and spot inoculations were then treated with either the bacteriophage (JG004 or P1), the disinfectant (sodium hypochlorite or benzalkonium chloride), or a combination of the phage and disinfectant applied sequentially (*n*_spot_ = 4, *n*_dry_ = 4, and *n*_wet_ = 5 under each test condition). All phage treatment mixtures were incubated at 37°C for 4 h, except for the extended dry biofilm treatments, which were incubated for up to 8 h. Phage solutions (10^2^ to 10^9^ PFU/ml) were prepared in LB medium to ensure a nutrient-rich environment for efficient phage infection. Volumes of phage preparations applied for each test setup were a spot inoculation volume (*V*_spot_) of 20 μl, *V*_dry_ of 1 ml, and *V*_wet_ of 200 μl. After phage treatment, biofilms were washed twice with PBS, while for the remaining spots, the liquid was carefully pipetted off before proceeding to sample evaluation or disinfectant treatment. All disinfectants were prepared and applied in PBS for 10 min at room temperature (5 to 200 ppm sodium hypochlorite and 5 to 200 ppm benzalkonium chloride), followed by solution removal and the application of a neutralizing agent for 10 min (1% sodium thiosulfate for sodium hypochlorite treatment or D/E neutralizing broth [BD, Heidelberg, Germany] for benzalkonium chloride treatment). Benzalkonium chloride was evaluated only in the spot inoculation experiments. After disinfectant quenching, biofilms were washed twice with PBS, while for the remaining spots, the liquid was carefully pipetted off before proceeding to sample evaluation. Untreated positive controls received either LB medium (during phage treatment) or PBS (during disinfectant treatment). For wet biofilms, the order of the combination treatment was explored by sequentially applying phage and then disinfectant as well as applying disinfectant followed by phage. Treatments were applied sequentially instead of simultaneously to prevent disinfectants from inactivating the phages.

### Recovery and evaluation of surface-attached bacteria.

After treatment, the remaining bacteria were recovered from spot inoculations and dry biofilms by placing the coverslips into 15-ml Falcon tubes (Fisher Scientific, Waltham, MA, USA) containing 5 ml of PBS. The tubes were vortexed at maximum speed for 1 min to detach cells from the coverslip. Similarly, wet biofilms were preserved in 200 μl PBS, and biofilms were detached from the well surface by manual scraping with a wooden applicator stick (Sigma-Aldrich, St. Louis, MO, USA) for 5 s each to remove attached biofilms. The well contents were then pipetted to resuspend the cells and break apart cell clumps.

### Flow cytometry.

All samples were evaluated for ICCs on an Accuri C6 sampler flow cytometer (FCM) (BD Biosciences, San Jose, CA, USA). Samples were stained with SYBR green I (SG) stain (Invitrogen AG, Basel, Switzerland) (100-fold diluted in 10 mM Tris buffer [pH 8]) at a final concentration of 1× as well as propidium iodide (final concentration of 1 × 10^6^ μM) to suppress fluorescence signals from cells that had been damaged and 5 mM EDTA to improve clustering. Samples were incubated for 10 min at 37°C to allow staining of the samples. Samples were then resuspended by pipetting or vortexing before measurement. Samples were appropriately diluted so that the measurements did not exceed 3,000 events/μl. All samples were prepared in Evian bottled water that had been filtered through a 0.1-μm filter to eliminate background bacteria (Millex VV Durapore polyvinylidene difluoride [PVDF] membrane). Fifty microliters of each sample was run on the FCM for measurement. Individual gates were drawn for each experimental setup to discriminate background signals from cells and used throughout these experiments. If the total number of events detected within the gate was <50, the value of that sample was set to 50 as the limit of quantification of the FCM (corresponding to a coefficient of variation [CV] of 15%) (75). This higher percent CV was determined to be allowable since the experiments were conducted with a single bacterial species and rare-event capture was not necessary. Individual measurements were normalized to the total for each sample (per well) and multiplied by the dilution factor to obtain the raw data. Data were then log transformed, and LRVs for each treatment were calculated by subtracting the log-transformed cell counts by the average of the corresponding positive-control log-transformed cell counts.

### Growth curves.

Growth curves were completed to determine the effect of drying on biofilm growth characteristics (lag time, population capacity, and growth rate). After biofilms were formed as described above, half of the biofilms were recovered before drying, while the other half were dried as described above. The biofilms were recovered and resuspended in TSB instead of PBS and used to fill 3 wells each on a microwell plate. The samples were incubated at 37°C under continuous shaking, and the cell density was inferred based on the optical density measured using the absorbance at 600 nm every 2 min for 20 h on an Eon microplate spectrophotometer (BioTek Instruments, Inc., Winooski, VT, USA).

### Phage inactivation by disinfectants.

Phage inactivation by disinfectants was assessed to determine the impact of disinfectants on phages and inform the extent to which disinfectant treatment following phage treatment would influence the infectivity of the phage. To investigate the effect of the disinfectants on the phages, phage P1 or JG004 (initial concentration of 1 × 10^8^ PFU/ml) was combined with various concentrations of sodium hypochlorite (to a final concentration of 5, 10, 15, 20, 50, 100, or 200 ppm) or benzalkonium chloride (to a final concentration of 5, 10, 20, 50, 100, or 200 ppm). Reactions were carried out for 1 min before being quenched with the appropriate neutralizing agent. Phage concentrations were then enumerated by a double-agar-layer assay (76) to determine log removal as a function of the CT (concentration × time) value.

### Statistical analyses.

Statistical analysis was performed in GraphPad Prism 8 (GraphPad, La Jolla, CA, USA) on log-transformed data normalized to the positive controls. Graphs were drawn in GraphPad Prism 8 using averages and standard deviations of data sets. Outliers were identified using “identify outliers” analysis, choosing the ROUT method with a *Q* value of 1% (77). One-way (phage-only or disinfectant-only experiments) or two-way (combination experiments) analysis of variance (ANOVA) was used to determine statistical differences between the applied treatments and control treatments. Multiple pairwise comparisons were corrected for using Tukey 95% confidence intervals.

The calculation of growth curve parameters was completed using R statistical software version 3.6.1 for OS X (R Core Team, Vienna, Austria [www.R-project.org]) within the RStudio integrated development environment. Growth characteristics, including the carrying capacity (*K*), initial population size (*N*_0_), and growth rate (*r*), were estimated using the Growthcurver R package (78). The lag-phase duration (λ) was estimated as the time of the intersection point of the horizontal line going through the initial absorbance, with the straight line describing the exponential growth phase. The initial absorbance was estimated as the average of the first 10 absorbance rates measured. The straight line describing the exponential growth phase was approximated as a tangent fitted to the inflection point of the logistic curve (occurs at half of the carrying capacity, obtained from the Growthcurver R package). This calculation of λ is a common approximation used for lag-phase description (79, 80). With average values of *K*, *N*_0_, *r*, and λ under each condition, *t* tests for growth curve comparisons were carried out in GraphPad Prism.

### Data availability.

All data have been deposited in the Eawag Research Data Institutional Collection (ERIC) for public availability at https://doi.org/10.25678/0004S5.
