# Supplementary material for: Bacteriophage Treatment before Chemical Disinfection Can Enhance Removal of Plastic-Surface-Associated Pseudomonas aeruginosa
Source: Appl Environ Microbiol. 2021 Sep 28;87(20):e00980-21. doi: 10.1128/AEM.00980-21 (PMC8478462; doi:10.1128/AEM.00980-21)
Supplement: Supplemental file 1 — Figures S1 to S4. Download AEM.00980-21-s0001.pdf, PDF file, 0.1 MB [file aem.00980-21-s0001.pdf]

Combined effect of bacteriophage treatment and chemical disinfection on surface-associated *Pseudomonas aeruginosa*

Elyse Stachler[a*], **Anina Kull**[a], **Timothy R. Julian**[a,b,c]

[a] Eawag, Swiss Federal Institute of Aquatic Science and Technology, CH-8600, Dübendorf, Switzerland

[b]Swiss Tropical and Public Health Institute, CH-4051 Basel, Switzerland

[c]University of Basel, CH-4055, Basel, Switzerland

* **Corresponding Author:** Elyse Stachler, Elyse.Stachler@eawag.ch, +41 58 765 6685

Pages: 4

Figures: 2

Figure S1: Comparison of growth curves of bacteria recovered from biofilms before and after drying.

Figure S2: Log reduction of *P. aeruginosa* in dry biofilms as a function of phage concentration.

Figure S3: Effect of initial chlorine treatment on subsequent phage treatment for phage P1.

Figure S4: Inactivation of phage JG004 and P1 by sodium hypochlorite and benzalkonium chloride.

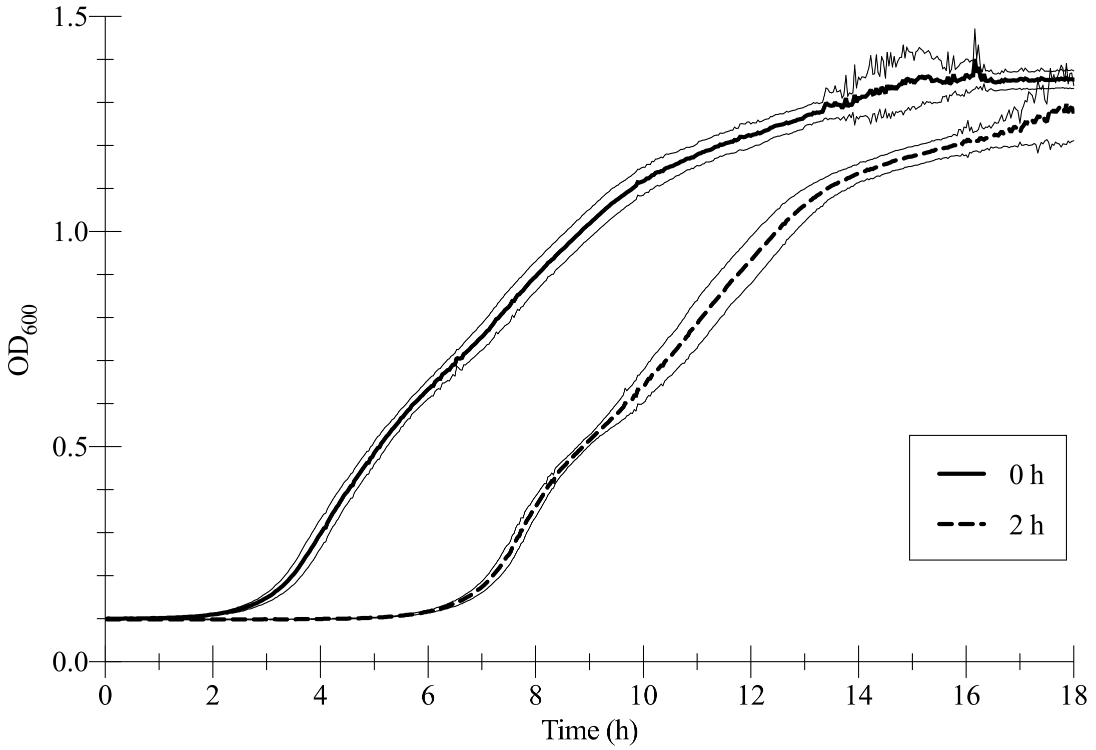

Figure S1: *Comparison of growth curves of P. aeruginosa recovered from biofilms that had been dried for 2 hours versus not being dried (0h).*

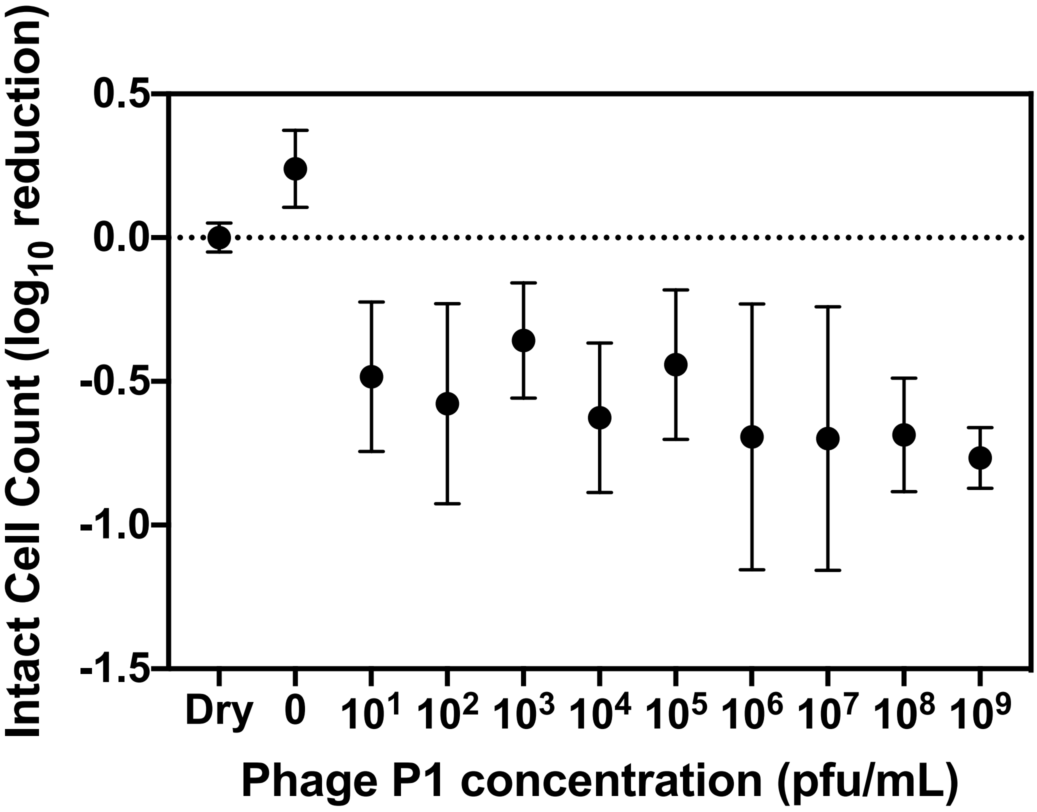

Figure S2: Log reduction of* P. aeruginosa *in dry biofilms. The data represents the average and standard deviation of four replicates of the log_{10} reduction of the Intact Cell Count of *P. aeruginosa* treated by 10^1-10^9 pfu/mL of phage P1 for 8 hours compared with no-treatment control biofilms.

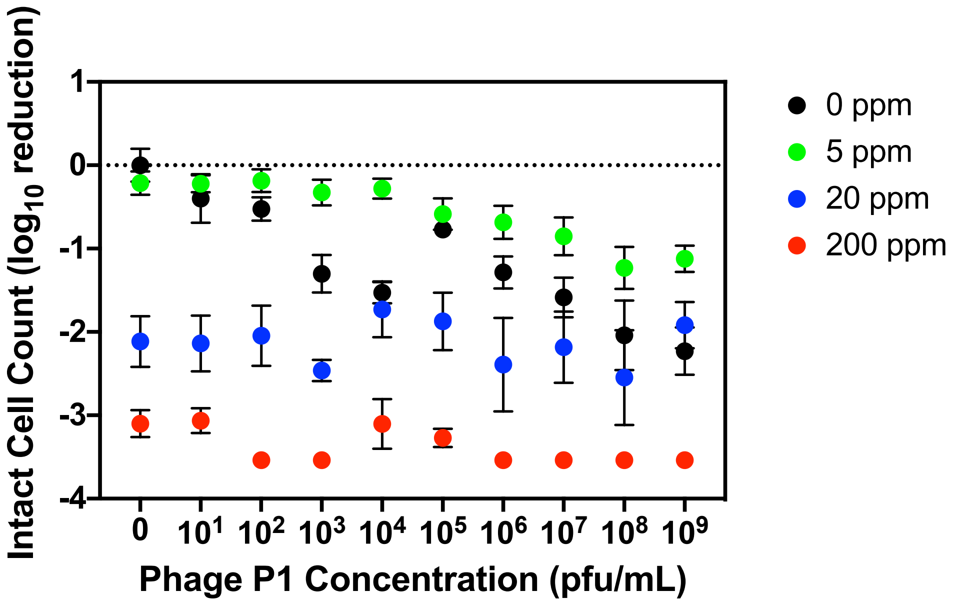

Figure 3: Effect of initial chlorine treatment on subsequential phage treatment for phage P1.

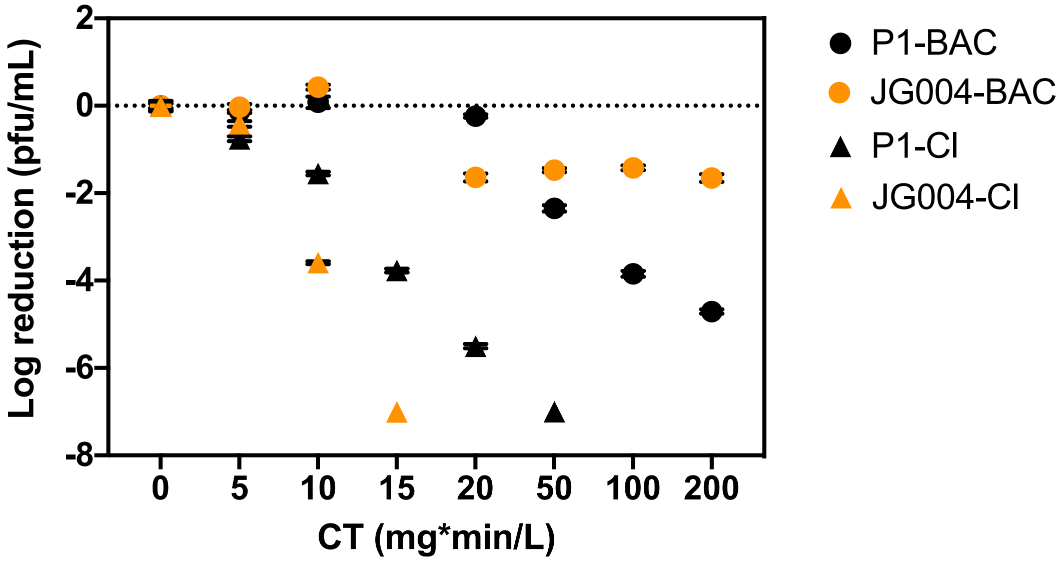

Figure 4: Inactivation of phage JG004 and P1 by sodium hypochlorite (Cl) and benzalkonium chloride (BAC).
